# Prof. Pasteur and the Yellow Fever

**Published:** 1882-03

**Authors:** 


### PROF. PASTEUR AND THE YELLOW FEVER.

Of Prof. Pasteur's journey in quest of yellow fever germs, M. Latour (*Union Medical*, September 24) writes as follows: Prof. Pasteur, who is not in early life, for he was born in 1822, and whose health has suffered from a serious accident, has in the midst of his brilliant career and all the celebrity his admirable discoveries have bestowed on his name, and when he ought to be enjoying some of that repose so legitimately acquired, given up his holidays and the vivifying air of his Jura mountains to go—where? To shut himself in a lazaretto in company with some unfortunate beings who have brought with them the fearful yellow fever from Senegal, in order to search in their dejections at the peril of his life for the *microbe* that may perhaps be the cause of this terrible affection, and in hope of being able by his skillful and patient "culture" to find the "vaccinator" for the black vomit as he has found it for anthrax and chicken-cholera. Whatever may be the result of his expedition it will do none the less honor to Prof. Pasteur. Even if his generous hopes are not realized he will at all events have given proof of his great courage in having undertaken these perilous experiments on a disease which by infection or contagion prostrates the most robust.—*Med. Times and Gaz.*

